# Post-trauma exophthalmos caused by a carotid-cavernous fistula

**DOI:** 10.1016/S1808-8694(15)30533-4

**Published:** 2015-10-18

**Authors:** Belmiro Cavalcanti do Egito Vasconcelos, Gabriela Granja Porto, Suzana Célia de Aguiar Soares Carneiro

**Affiliations:** 1 PhD, Graduate Program Coordinator at UPE; 2 MSc in Oral and Maxillofacial Surgery and Trauma, Doctoral student in Oral and Maxillofacial Surgery and Trauma; 3 MSc in Oral and Maxillofacial Surgery and Trauma, Doctoral student in Oral and Maxillofacial Surgery and Trauma

**Keywords:** exophthalmos, wounds, carotid-cavernous, fistula, injuries

## INTRODUCTION

Fistulae may be defined as direct connections between arteries and veins^1^. Carotid-cavernous fistulae (CCF) are formed secondarily to abnormal communications between the cavernous portion of the carotid artery and the venous plexus of the cavernous sinus^1^, ^2^, ^3^.

Traumatic fistulae usually result from injuries to the internal carotid artery in its course to the cavernous sinus^4^. These types of fistula frequently appear a few weeks after trauma with signs and symptoms related to increased venous pressure transmitted through the ophthalmic vein, a valve-free vessel^2^.

This paper presents the means to diagnose carotid-cavernous fistulae and a case report.

## CASE PRESENTATION

**Figure 1:**
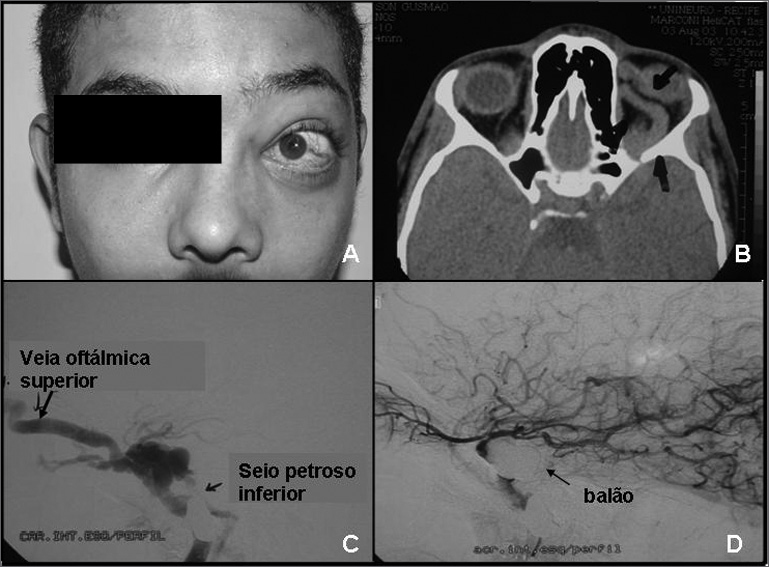
(A-D) - A - Proptosis; B - CT scan reveals much dilated superior ophthlalmic vein; C - brain digital angiography; D - carotid-cavernous fistula balloon embolization.

A 17-year-old male was injured in his face as a result of an accident with a firearm. Initial treatment consisted of conservative debridement; the wound was cleaned and soft tissue sutured. Fifteen days after the accident the patient came back to our service complaining of left eye pain and edema in the ipsilateral orbit. Physical examination revealed proptosis and limited mobility of the left eye (Fig. 1A). Murmur was observed in the left orbit. No cranial nerve deficit was identified. Before such clinical findings, the patient was diagnosed with carotid-cavernous fistula (CCF) and was referred to the ophthalmologist for further examination and vascular therapy. Contrast-enhanced CT scans showed a much dilated superior ophthalmic vein suggestive of fistula (Fig. 1B). Brain digital angiography revealed the presence of a post-traumatic CCF with direct cavernous sinus opacification and filling of the superior ophthalmic vein (Fig. 1C). CCF balloon embolization was subsequently attempted by a vascular surgeon using the bifemoral approach with no success.

A microcatheter was then passed through the right vertebral artery. The device was navigated from the left posterior communicating artery, anteriorly to the intracavernous position and distally to the left carotid; the fistula was embolized with detachable coils and completely removed using this approach (Fig. 1D). Three weeks into treatment both the fistula and the proptosis regressed. Orbit murmur could no longer be observed. The patient recovered from the symptoms and remained free of neurologic deficit.

## DISCUSSION

Carotid-cavernous fistulae are rare trauma complications^3^. Incidence rates range between 0.2-0.3% in all cases of head and face trauma^5^. Pulsating exophthalmia, orbit murmur, conjunctival hyposphagma, ophthalmoplegia, eye pain, and reduced visual acuity are the most frequently observed signs and symptoms^5^. Our patient presented ecchymosis, limited eye mobility, eye pain, proptosis, and orbit murmur, as also found in the literature.

There is consensus in the literature^2^,^3^,^4^ over the use of transarterial balloons to treat CCF. Ideally, fistulae should be embolized at the time the injury is diagnosed^5^, as performed in our case.

The size of both cavernous sinus and fistula may affect the CCF embolization success rate6. The cavernous sinus must be broad enough to accommodate the embolization balloon. The fistula should be smaller than the inflated balloon, yet ample enough to allow the placement of a fully or partially inflated balloon^6^. Our patient was successfully treated as there was a broad connection of the fistula between the branches of the external carotid artery and the cavernous sinus.

## CONCLUSION

Carotid-cavernous fistulae are rarely seen in the daily practice of oral and maxillofacial surgery. Although they may appear at a later time secondary to face trauma, oral and maxillofacial surgeons must pay close attention to the early symptoms reported by the patient and progressive ipsilateral orbit edema at 15 days after trauma.
